# Socio-economic factors associated with post-traumatic stress symptoms among adolescents and young people during the first wave of the COVID-19 pandemic

**DOI:** 10.1038/s41598-023-50333-8

**Published:** 2024-01-27

**Authors:** Morenike Oluwatoyin Folayan, Roberto Ariel Abeldaño Zuñiga, Passent Ellakany, Muhammad Abrar Yousaf, Bamidele Emmanuel Osamika, Jorma I. Virtanen, Balgis Gaffar, Folake Barakat Lawal, Zumama Khalid, Nourhan M. Aly, Joanne Lusher, Annie Lu Nguyen

**Affiliations:** 1 Mental Health and Wellness Study Group, Obafemi Awolowo University, Ile-Ife, Nigeria; 2 Department of Child Dental Health, Obafemi Awolowo University, Ile-Ife, Nigeria; 3 Postgraduate Department, University of Sierra Sur, Oaxaca, Mexico; 4 Department of Substitutive Dental Sciences, College of Dentistry, Imam Abdulrahman Bin Faisal University, Dammam, Saudi Arabia; 5 Department of Biology, Faculty of Science and Technology, Virtual University of Pakistan, Lahore, Pakistan; 6 Department of Psychology and Institute for the Environment and Sustainability, Miami University, Oxford, OH USA; 7 Faculty of Medicine, University of Turku, Turku, Finland; 8 Department of Preventive Dental Sciences, College of Dentistry, Imam Abdulrahman Bin Faisal University, Dammam, Saudi Arabia; 9 Department of Periodontology and Community Dentistry, University of Ibadan and University College Hospital, Ibadan, Nigeria; 10 Department of Health Sciences, University of Genova, 16132 Genoa, Italy; 11 Department of Pediatric Dentistry and Dental Public Health, Faculty of Dentistry, Alexandria University, Alexandria, Egypt; 12 Provost’s Group, Regent’s University London, London, UK; 13 Department of Family Medicine, Keck School of Medicine, University of Southern California, Los Angeles, USA

**Keywords:** Diseases, Risk factors

## Abstract

This study assessed the association between sociodemographic factors and post-traumatic stress symptoms (PTSS) among 18–24-year-olds during the first wave of the COVID-19 pandemic. This was a secondary analysis of data from 4508 individuals collected through an online survey conducted between June and January 2021. PTSS was measured as a dependent variable using the checklist for post-traumatic stress disorder in civilians. Age, birth sex, sexual, level of education, access to emotional and social support, and emotional distress were the independent variables. A multivariate logistic regression analysis was conducted to determine the associations between the dependent and independent variables while controlling for the country related confounding variables. Females (AOR:2.023), sexual minority individuals (AOR:1.868), those who did not disclose their sexual identify (AOR:1.476), those with poor access to emotional and social support (AOR:4.699) and individuals with no formal education (AOR:13.908), and only primary level education (AOR:4.521) had higher odds of PTSS. The study highlights the multifaceted nature of PTSS during the pandemic and suggests the importance of promoting access of young people, especially females, sexual minority individuals and those with low educational status, to emotional/social support to mitigate the probability of PTSS, especially among sexual minority individuals.

## Introduction

The COVID-19 pandemic, described as a potentially traumatic event due to its unpredictable, severe, and prolonged nature, as well as the unfamiliar and life-threatening aspects it presents^1^, has been linked to symptoms of post-traumatic stress^2–4^. Some individuals face an elevated risk of developing post-traumatic stress symptoms (PTSS) during the pandemic. This includes those who experienced a severe bout of COVID-19, bore witness to the suffering and loss of others due to COVID-19, received news of the death or impending danger to a family member or friend, and those who had extensive exposure to adverse events^1^.

The prevalence of PTSS ranges from over 17% in individuals with COVID-19^5^, to 16% in survivors of severe COVID-19^5^, 17% within the general population^6^, 21.5% among healthcare professionals^7,8^ and 23% in Vietnam during the fourth wave of COVID-19^9^. However, there is limited information available regarding the prevalence of PTSS among adolescents’ and young individuals, despite substantial evidence indicating that the COVID-19 pandemic had a negative effect on the mental health of adolescents and young people^10–12^. One such study conducted in Turkey indicated that as high as 28.5% of adolescents had COVID-19 induced PTSS^13^. Another study conducted in UK, indicated that the prevalence of PTSS among adolescents and young people was 29%^14^, while a prevalence of 21.1% was reported among students globally^15^.

Not only is there limited information on the prevalence of PTSS among various populations of adolescents and young people, but there is also a shortage of literature concerning factors that increase the risk for PTSS in this population. The risk of developing PTSS may be higher for females^16^, for those who are younger^17^ and individuals with lower levels of education^18^. In addition, exposure to COVID-19 has been shown to increase the risk for PTSS^19^, whilst access to emotional and social support can reduce the risk for PTSS^14^.

Furthermore, marginalized sexual minority groups (gay, lesbian, and bisexual individuals), can face an increased susceptibility to PTSS due to persistent exposure to societal stressors^20–22^. However, there remains a gap in understanding of how COVID-19 may have differentially impacted the risk of PTSS among sexual minority adolescents and young adults. Although previous research has suggested a higher risk of mental health issues, including post-traumatic stress disorders, among sexual minority individuals compared to heterosexual individuals during the pandemic^23^, there is emerging evidence indicating potential age-related variations in the mental health experiences of sexual minority groups^24,25^. It is therefore imperative to further understand how the risk of PTSS varies for young people based on their sexual orientation. Studying PTSS is essential here as while PTSS may be transient, it is a known avenue for post-traumatic stress disorder. If left untreated, post-traumatic stress disorder increases the risk for long-term poor physical and psychological health^26^. Access to emotional and social support, however, reduces the risk for PTSS among adolescents and young people^27^, thus amplifying the importance of identifying risk indicators for PTSS among adolescents and young people during the COVID-19 pandemic.

The present study was further prompted by the understanding that vulnerability to PTSS can be influenced by psychosocial and economic hardship resulting in emotional distress. In this study, we employed the PTSS learning model to uncover psychological consequences of the pandemic that could lead to PTSS in young individuals^26^. Our hypothesis was that difficulty in adapting to previous traumatic experiences may be a causative factor for experiencing fear and negative emotions associated with COVID-19. The conditioning process contributes to re-experiencing distress triggered by exposure to stressful stimuli (such as COVID-19) that resembles aspects of past trauma. In this study, we assessed PTSS among young adults at risk of having been exposed to prior adverse events (such as those with sexual minority status, people living with HIV, and individuals in lower socioeconomic status). The primary objective of this study was to identify associations between age, gender, sexual identity, education level, access to emotional and social support and PTSS among young adults during the first wave of the COVID-19 pandemic. It was hypothesized that adolescents and young people, females, sexual minority individuals, those with lower educational status and those with poor access to emotional and social support would be at increased risk of experiencing PTSS.

## Methods

### Ethical considerations

The primary study received ethical approval from the Human Research Ethics Committee at the Institute of Public Health, Obafemi Awolowo University, Ile-Ife, Nigeria (HREC No: IPHOAU/12/1557). Additionally, ethical clearance was granted in Brazil (CAAE N° 38423820.2.0000.0010), India (D-1791-uz and D-1790-uz), Saudi Arabia (CODJU-2006F), and the United Kingdom (13283/10570). All procedures and methodologies adhered to applicable guidelines and regulatory standards. All participants provided written informed consent before taking the survey.

### Study design and study population

This secondary analysis involved using data from an online survey conducted in 152 countries between June and January 2021 as part of a cross-sectional study. Detailed procedures for conducting this survey are documented elsewhere^28–30^ with details concerning the validation of the questionnaire^28^. The survey compiled responses from individuals aged 18 years and above who voluntarily provided their consent to participate in the study that assessed risk indicators for COVID-19 related mental health and wellness. No specific exclusion criteria were applied.

For this analysis, data from 4508 adolescents and young adults aged 18 to 24 years from 105 countries (see Appendix 1), accounting for 21.4% of the total dataset of 21,106 respondents, were extracted. The extracted dataset was deemed suitable for statistical modelling, as it facilitated the execution of regression analyses with a minimum significance level (p-value) of 0.05^31^.

### Participant recruitment

The primary study recruited participants using a convenient sampling technique. The recruitment of participants was achieved through the dissemination of the online survey link on various social media platforms including Facebook, Twitter, and Instagram, as well as through network email lists and WhatsApp groups.

Data collection was conducted anonymously. The privacy of participants and the confidentiality of the information they provided were safeguarded by disassociating the IP addresses from the questionnaire upon completion of the online survey. Furthermore, the questionnaire did not employ any tracker cookies that could potentially be installed on the respondents' devices. Data collection was facilitated using SurveyMonkey^®^, a platform that ensures a secure, SSL-encrypted connection. Data were encrypted using robust TLS cryptographic protocols. SurveyMonkey^®^ is certified for compliance with both the EU-U.S. Privacy Shield Framework and the Swiss-U.S. Privacy Shield.

### Data collection tool

The study questionnaire was originally created in English and subsequently translated into French, Spanish, Arabic, and Portuguese. To verify the consistency of the content, the French, Spanish, Arabic, and Portuguese versions were back translated into English. The questionnaire underwent a comprehensive assessment, including evaluations of validity, dimensionality, and reliability, as well as a qualitative assessment. The overall content validation index for the study questionnaire stood at 0.83. Detailed information regarding the validation of the data collection tool can be found in a published source^28^. Further details are available in the same publication^28^. Additional information about the data collection tools can be found elsewhere^28–30^.

### Study dependent variable

The dependent variable of interest was PTSS, which was assessed using the 17-item self-reported checklist for post-traumatic stress disorder in civilians^32^. This checklist employs a 5-point Likert scale, where responses range from 1 (not at all) to 5 (extremely). The possible scores on this scale span from 17 to 85. To categorize the responses, a cut-off point of 28 was applied, effectively dichotomizing the outcomes into 'no PTSS' (scores between 17 and 27) and 'PTSS present' (scores between 28 and 85)^33^. The measure's specific details have been previously documented in two prior studies extracted from the database^14,34^. In terms of the instrument's test reliability, the intra-class correlation coefficient was calculated to be 0.89, indicating excellent reliability^28^. Additionally, the content validity index for the instrument was established at 0.83^28^. Though the tool can be used to screen for post-traumatic stress disorder, clinical confirmation was not carried out as part of this study, so the variable was treated as PTSS.

### Study independent variables

The independent variables included age, which was categorized into two groups: < 20 years (adolescents) and 20–24 years (young adults); sex at birth (male, female); sexual identity, classified as heterosexual or gay, lesbian, and bisexual individuals (considered as sexual minority); level of education (ranging from no formal education to primary, secondary, and college/university); and access to emotional support from friends and family. To measure access to emotional and social support, respondents were asked to indicate if they received emotional or social support from family, friends, partners, counsellors, or others by checking an appropriate response. This question was extracted from the Pandemic Stress Index^35^.

### Study confounding variable

Country income level was a variable that could potentially introduce confounding effects. This is because the income level of countries can influence the prevalence and severity of traumatic events that individuals are exposed to^36^ and this influence can differ by sociodemographic profile^36^. The level of access to support systems can also differ by country income-level^37^. Country income level was derived from publicly accessible data from the World Bank Data Bank^38^. Countries were categorized based on their income level into the following groups: low‐income countries (LICs) with a gross national income (GNI) per capita of ≤ 1035 USD in 2019, lower middle‐income countries (LMICs) with GNI falling between 1036 and 4045 USD, upper middle‐income countries (UMICs) with GNI ranging from 4046 to 12,535 USD, and high‐income countries (HICs) with a GNI of ≥ 12,536 USD.

**Figure 1:**
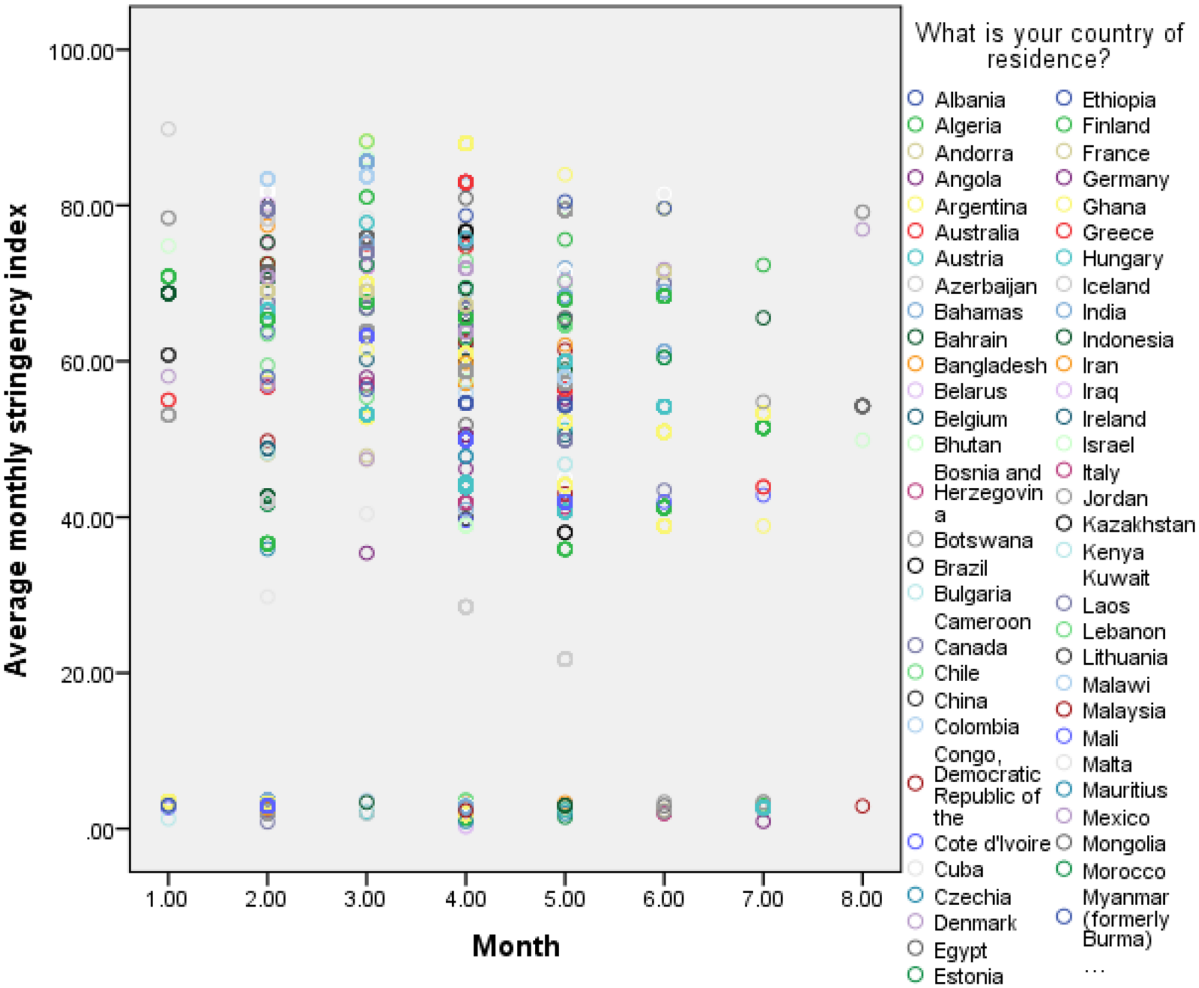
Scatterplot of monthly average stringency index by country: June 2020 to January 2021.

In addition, the pandemic stringency index was included to capture the impact of government policies related to closure and containment, health, and economic policy^39^. The index includes policy responses in 19 policy areas, capturing variation in degree of response. For this study, we calculated and imputed the average of the stringency index calculated for the participants’ country of residence, during the month in which respondents completed the survey. The index ranges from 0 to 100, and according to the authors, the higher the index, the harder the stringency of the covid policies for a given country. We categorized the variable into an ordinal scale (0–19.99, 20–39.99, 40–59.99, 60–79.99, and 80–100)^39^. Figure 1 shows the dynamics of this stringency index by month, for each country of residence of the participants, being June 2020 the first month, and January 2021 the eighth month.

### Data analysis

The data underwent a thorough cleaning process and was subsequently imported into SPSS version 23.0 (IBM Corp., Armonk, N.Y., USA) for further analysis. We compared the groups with and without PTSS using chi square and t-test where appropriate. A multivariate logistic regression analysis was performed to assess determine the sociodemographic variables that were risk indicators for PTSS. This analysis examined the relationships between the dependent and independent variables while accounting for the influence of the confounding variable. Adjusted odds ratios (AOR) and their corresponding 95% confidence intervals (CI) were calculated. The level of statistical significance was set at 5%. The missing data were treated through multiple imputation techniques for the following variables: sex (1.0% of missing values), country of residence (12.2% of missing values), sex identity (16.5% of missing values), Average stringency index (16.3% of missing values) and PTSS (18.0% of missing values). For continuous variables (stringency index) we used the linear regression method, while for categorical variables we used the logistic regression method for imputation.

## Results

Of the 4508 respondents, the majority were from Pakistan (N = 722; 16.0%), Nigeria (N = 659; 14.6%) and India (N = 450; 10.0%) as shown in Appendix 1. Table 1 shows that 3201 (71.0%) respondents were female, 3788 (84.0%) were between 20 and 24 years, 2777 (61.6%) had college/university education, and 3481 (77.2%) were heterosexuals. In addition, 1525 (33.8%) had PTSS and 2375 (52.7%) had one or more types of emotional stress.

**Table 1:** Descriptive statistics and associated factors of post-traumatic stress symptoms in adolescents and young people recruited online from 105 countries (N = 4508).

Variables	Total N = 4508 n (%)	Post-traumatic stress symptoms		P value	AOR; 95% CI; p value
		Yes N = 1525 n (%)	No N = 2983 n (%)		
Age					
< 20 years	720 (16.0)	237 (32.9)	483 (67.1)	0.606	0.857; 0.711–1.032; p = 0.857 1.000
20–24 years	3788 (84.0)	1288 (34.0)	2500 (66.0)		
Sex at birth					
Female	3201 (71.0)	1222 (38.2)	1979 (61.8)	< 0.001	2.023; 1.729–2.367; p < 0.001 1.000
Male	1307 (29.0)	303 (23.2)	1004 (76.8)		
Sexual identity					
Heterosexual	3481 (77.2)	1063 (30.5)	2418 (69.5)	< 0.001	1.000 1.868; 1.452–2.402; p < 0.001 1.476; 1.229–1.773; p < 0.001
Sexual minority	309 (6.9)	148 (47.9)	161 (52.1)		
Undisclosed	718 (15.9)	314 (43.7)	404 (56.3)		
Level of education					
No formal education	92 (2.0)	81 (88.0)	11 (12.0)	< 0.001	13.908; 7.298–26.505; p < 0.001 4.521; 2.873–7.115; p < 0.001 1.131; 0.997–1.309; p = 0.098 1.000
Primary	96 (2.1)	64 (66.7)	32 (33.3)		
Secondary	1543 (34.2)	527 (34.2)	1016 (65.8)		
College/university	2777 (61.6)	853 (30.7)	1924 (69.3)		
Access to emotional and social support					
Yes	4023 (89.2)	1217 (30.3)	2806 (69.7)	< 0.001	1.000 4.699; 3.830–5.766; p < 0.001
No	485 (10.8)	308 (63.5)	177 (36.5)		

Age was not significantly associated with PTSS. However, females had higher odds of PTSS than males (AOR: 2.023; 1.729–2.367; p < 0.001). In addition, sexual minority individuals (AOR: 1.868; 1.452–2.402; p < 0.001) and those who did not disclose their sexual identity (AOR: 1.476; 1.229–1.773; p < 0.001) had higher odds of PTSS) had higher odds of PTSS than those heterosexual individuals. Furthermore, respondents who do not had access to emotional and social support also had higher odds of experiencing PTSS than those who had access to emotional and social support (AOR: 4.699; 3.830–5.766; p < 0.001). Respondents with no formal education (AOR: 13.908; 7.298–26.505; p < 0.001) and those with only primary education (AOR: 4.521; 2.873–7.115; p < 0.001) also had significantly higher odds of PTSS than those without a college/university level of education.

## Discussion

The study findings suggest that some factors may increase the probability of sub-populations of young people developing PTSS during the first wave of the COVID-19 pandemic. These subpopulations are being a female, being a sexual minority individual or one with undisclosed sexual identity, those with no formal education or only primary level of education, and those who had no access to emotional and social support. These results align with the study's initial hypotheses.

One strength of this research is its global reach, as the sample included adolescents and young individuals from various parts of the world. The study offers valuable insights into the mental health of young people during the first wave of the COVID-19 pandemic, particularly at a time when it was assumed that the virus had a milder biological effect on this demographic^40^. This study also contributes to the limited body of research on PTSS among adolescents and suggests that being young and from a sexual minority group increases the probability of having post-traumatic stress. This aligns with a previous study, which found that sexual minority young adults exhibit a 1.6- to 3.9-fold higher likelihood of probable post-traumatic stress disorder compared to their heterosexual counterparts^41^.

However, it's important to note that these results may not be generalizable due to the recruitment method, which involved a non-probability sampling technique, potentially introducing selection bias. Additionally, data collection occurred online, potentially excluding young individuals without internet access or smartphones. Those whose languages were not covered by the questionnaire may also have been inadvertently omitted. Furthermore, the number of participants in some of the countries from which data were extracted were very few and unevenly distributed. Also, the study was cross-sectional in nature, making it challenging to establish cause-and-effect relationships. The use of the checklist for post-traumatic stress disorder in civilians for screening for PTSS is likely to generate a higher prevalence of PTSS than when the post-traumatic stress disorder check list for DSM-5 is used. The diagnostic criteria for post-traumatic stress disorder had been modified to improve the accuracy and consistency^42^. Moreover, it is probable that the prevalence of PTSS could be overestimated, given that the study participants encompassed individuals beyond survivors of a traumatic event. Furthermore, the questions in the PTSD tool used in this study to measure PTSS exhibit a high correlation with general distress. As a result, the reported values of PTSS in this study may more accurately represent general distress rather than specifically capturing PTSS^43^. Despite these limitations, the study findings can offer valuable hypotheses for future research to examine.

These preliminary findings contribute to the limited literature on the impact of the COVID-19 pandemic on young adults. This current study suggests that, during the first wave of the pandemic, young sexual minority individuals were more likely to have PTSS than heterosexuals. Previous studies have also shown that more sexual minority individuals experienced severe mental health impacts during the pandemic compared to heterosexuals^23^. This study, therefore, adds to the existing body of evidence. In addition, however, the study results indicated that young people who did not disclose their sexual identity had a high probability of experiencing PTSS. Individuals who do not disclose their sexual identity are more likely to be bisexual individuals^44^ and are more likely to have poorer psychological wellbeing^44^. Our deviating from the standard norms of only comparing heterosexual and non-heterosexual study participants may have enabled us unobscure meaningful differences within the sexual minority population and identify a population of young individuals may need mental health care during and post-pandemics.

The results of the current study also suggest that young females exhibit higher rates of PTSS compared to young males and individuals with a higher educational level, which is line with previous studies^45,46^. The observed differences in PTSS susceptibility between genders might be linked to distinct brain activations. For example, males tend to activate the inferior parietal lobule when encountering abrupt or ambiguous events, while females show more activity in the postcentral gyrus^47^. This divergence may lead females into a cycle of acute emergency disorder that can eventually develop into PTSD. In contrast, males often exhibit quicker emotional control and a propensity to strategize their responses to events, while females are more likely to experience panic, limiting their ability to respond effectively^47^.

The gender related variation in PTSS susceptibility could also be due to hormonal fluctuations resulting from menstruation or highly stressful events. Notably, female sex hormones like progesterone and estradiol play a pivotal role in regulating mood states^48–50^. Additionally, females are more likely than males of similar age to experience interpersonal stressors, gender-based violence, gender inequality, and discrimination due to traditional cultural and social role differences^51^. Consequently, females are more prone to anxiety, depression, PTSS, and other mental health disorders. The implications of PTSS are significant, affecting various aspects of female college students' lives, including their future, employment, academic performance, and physical and mental well-being^52^. However, the results of meta-analysis are not conclusive: a meta-analysis pointed to a lack of difference in PTSD prevalence by gender^53^ while another showed gender differences^54^. These studies did not target adolescents and young individuals. There may be gender related differences in risk factors for PTSS by age, but without further evidence this remains inconclusive.

Furthermore, the observed higher probability of PTSS among adolescents and young individuals with lower educational status in the current study may be related to concerns about the future and job security, particularly among those with limited experience in navigating social media and understanding the effects of the pandemic on health and the economy^52,55^. Some other studies, however, suggest that higher educational attainment is associated with a higher risk of developing PTSD^56–58^ due to heightened awareness about the impact of COVID-19 on their health and potential post-infection complications^56^. Once again, the reasons for the observed disparity may be age related and the findings should be replicated.

Additionally, poor access to emotional or social support had a significant impact on the experience of PTSS among adolescents and young individuals in this study. Several studies have indicated that individuals living with their families or roommates during lockdown experienced lower levels of depression, anxiety, and PTSS^46,59^. Pandemics like COVID-19 can increase the risk of some populations to PTSS^53^. The increase observed among adolescents and young people may be due to the increased possibility of being dissociated from sources of emotional and social support during the pandemic.

For adolescents and young people, the absence of social support such as school and parental social support, may increase the possibility for mental health problems^60,61^. Adolescents and young people experience significant physical and emotional changes, and often navigate complex social dynamics. Adequate social support acts as a buffer against stressors, providing a sense of belonging and emotional security. The school environment, with its structured interactions and peer relationships, plays a pivotal role in shaping an adolescent's social experience^62^. Positive school support fosters a sense of community and can serve as a protective factor against mental health challenges^63^. Similarly, parental support is crucial during this period of exploration and identity formation. Adolescents rely on parental guidance and emotional backing as they navigate the challenges of transitioning into adulthood^63^. Moreover, adolescence represents a time for social networking, and positive interactions within these networks contribute to a sense of belonging and provide emotional sustenance, essential for coping with the inherent stressors of adolescence^64^. The absence of the robust social support may create the feelings of isolation potentially exacerbating mental health problems. Therefore, interventions and support systems that reinforce positive social connections and relationships are vital to promoting the mental health and well-being of adolescents and young people during pandemics like the COVID-19.

This study also demonstrates that, in addition to healthcare workers and those who contracted COVID-19, subpopulations of the public may also face an increased probability of having PTSS during the pandemic^65^. Moreover, the present findings suggest that females, young people with lower levels of education, individuals with no access to emotional and social support, and sexual minority individuals who are young people are more likely to experience PTSS due to the COVID-19 pandemic. Prior studies have indicated that adults with lower educational status are at a higher risk for PTSS, although the risk of PTSS increases with age and is significantly higher among females^66^. Given the focus on young adults in this study, age-related associations with PTSS may not have been detected. However, consistent with other studies, this research suggests that emotional and social support may reduce the probability of PTSS as indicated in prior research^67,68^. Emotional and social support can facilitate the use of positive coping strategies to address emotional stress, thereby reducing the PTSS^69^. The interplay of various factors influencing PTSS among young adults during the COVID-19 pandemic warrants further exploration.

In conclusion, this study suggests that sociodemographic factors, excluding age, may be linked to PTSS in adolescents and young individuals during the wave of the COVID-19 pandemic. It further demonstrates that sexual minority individuals who are young and those exposed to multiple forms of emotional distress may be more prone to PTSS, while access to social and emotional support seems to mitigate this susceptibility. Promoting access to sources of social and emotional support for adolescents and young people during the COVID-19 pandemic, as well as future pandemics with similar characteristics, may be crucial in reducing exposure to PTSS. Future research might wish to further explore how individual and country-level factors moderated these associations between sociodemographic factors and PTSD in adolescents and young individuals during the COVID-19 pandemic.

### Supplementary Information

Supplementary Information.

## Data Availability

The datasets used and/or analysed during the current study are available from the corresponding author on reasonable request.
