# Supplementary material for: Socio-economic factors associated with post-traumatic stress symptoms among adolescents and young people during the first wave of the COVID-19 pandemic
Source: Sci Rep. 2024 Jan 27;14:2276. doi: 10.1038/s41598-023-50333-8 (PMC10821943; doi:10.1038/s41598-023-50333-8)
Supplement: Supplementary file 1 — Supplementary Information. [file 41598_2023_50333_MOESM1_ESM.docx]

Appendix 1

Distribution of respondents by country

| Country of residence | n | % |
| --- | --- | --- |
| Albania | 2 | 0.0 |
| Algeria | 6 | 0.1 |
| Andorra | 2 | 0.0 |
| Angola | 2 | 0.0 |
| Argentina | 96 | 2.1 |
| Australia | 6 | 0.1 |
| Austria | 1 | 0.0 |
| Azerbaijan | 1 | 0.0 |
| Bahamas | 1 | 0.0 |
| Bahrain | 7 | 0.2 |
| Bangladesh | 3 | 0.1 |
| Belarus | 2 | 0.0 |
| Belgium | 2 | 0.0 |
| Bhutan | 1 | 0.0 |
| Bosnia and Herzegovina | 25 | 0.6 |
| Botswana | 1 | 0.0 |
| Brazil | 6 | 0.1 |
| Bulgaria | 1 | 0.0 |
| Cameroon | 2 | 0.0 |
| Canada | 13 | 0.3 |
| Chile | 4 | 0.1 |
| China | 3 | 0,1 |
| Colombia | 2 | 0.0 |
| Congo, Democratic Republic of the | 2 | 0.0 |
| Cote d'Ivoire | 1 | 0.0 |
| Cuba | 1 | 0.0 |
| Czechia | 1 | 0.0 |
| Denmark | 1 | 0.0 |
| Egypt | 177 | 3.9 |
| Estonia | 1 | 0.0 |
| Ethiopia | 1 | 0.0 |
| Finland | 108 | 2.4 |
| France | 3 | 0.1 |
| Germany | 9 | 0.2 |
| Ghana | 73 | 1.6 |
| Greece | 6 | 0.1 |
| Hungary | 70 | 1.6 |
| Iceland | 2 | 0.0 |
| India | 450 | 10.0 |
| Indonesia | 21 | 0.5 |
| Iran | 6 | 0.1 |
| Iraq | 2 | 0.0 |
| Ireland | 2 | 0.0 |
| Israel | 7 | 0.2 |
| Italy | 2 | 0.0 |
| Jordan | 254 | 5.6 |
| Kazakhstan | 3 | 0.1 |
| Kenya | 3 | 0.1 |
| Kosovo | 1 | 0.0 |
| Kuwait | 81 | 1.8 |
| Laos | 1 | 0.0 |
| Lebanon | 4 | 0.1 |
| Lithuania | 2 | 0.0 |
| Malawi | 1 | 0.0 |
| Malaysia | 4 | 0.1 |
| Mali | 2 | 0.0 |
| Malta | 1 | 0.0 |
| Mauritius | 1 | 0.0 |
| Mexico | 183 | 4.1 |
| Mongolia | 1 | 0.0 |
| Morocco | 6 | 0.1 |
| Myanmar (formerly Burma) | 2 | 0.0 |
| Namibia | 1 | 0.0 |
| Netherlands | 1 | 0.0 |
| New Zealand | 2 | 0.0 |
| Nigeria | 659 | 14.6 |
| Oman | 2 | 0.0 |
| Pakistan | 722 | 16.0 |
| Palestine | 22 | 0.5 |
| Peru | 8 | 0.2 |
| Philippines | 311 | 6.9 |
| Poland | 3 | 0.1 |
| Portugal | 3 | 0.1 |
| Qatar | 2 | 0.0 |
| Romania | 3 | 0.1 |
| Russia | 7 | 0.2 |
| Rwanda | 1 | 0.0 |
| Saudi Arabia | 305 | 6.8 |
| Senegal | 2 | 0.0 |
| Serbia | 2 | 0.0 |
| Slovenia | 1 | 0.0 |
| South Africa | 111 | 2.5 |
| Spain | 19 | 0.4 |
| Sri Lanka | 3 | 0.1 |
| Sudan | 36 | 0.8 |
| Sweden | 2 | 0.0 |
| Syria | 241 | 5.3 |
| Taiwan | 1 | 0.0 |
| Tanzania | 1 | 0.0 |
| Thailand | 3 | 0.1 |
| Turkey | 13 | 0.3 |
| Uganda | 2 | 0.0 |
| Ukraine | 5 | 0.1 |
| United Arab Emirates (UAE) | 20 | 0.4 |
| United Kingdom (UK) | 65 | 1.4 |
| United States of America (USA) | 37 | 0.8 |
| Uruguay | 1 | 0.0 |
| Uzbekistan | 1 | 0.0 |
| Venezuela | 21 | 0.5 |
| Vietnam | 4 | 0.1 |
| Yemen | 174 | 3.9 |
| Zambia | 1 | 0.0 |
| Zimbabwe | 4 | 0.1 |
| Samoa - American | 1 | 0.0 |
| Total | 4508 | 100.0 |
